# Trends in socioeconomic inequalities among adult male hardcore smokers in Vietnam: 2010–2015

**DOI:** 10.1186/s12939-017-0623-x

**Published:** 2017-07-14

**Authors:** Vu Duy Kien, Tej Ram Jat, Kim Bao Giang, Phan Thi Hai, Doan Thi Thu Huyen, Luong Ngoc Khue, Nguyen Tuan Lam, Phan Thi Quynh Nga, Nguyen The Quan, Hoang Van Minh

**Affiliations:** 1 Center for Population Health Sciences, Hanoi School of Public Health, Hanoi, Vietnam; 2 HelpAge International, Yangon, Myanmar; 3 Institute for Preventive Medicine and Public Health, Hanoi Medical University, Hanoi, Vietnam; 4 Vietnam Steering Committee on Smoking and Health (VINACOSH), Hanoi, Vietnam; 5 World Health Organization Office in Viet Nam, Hanoi, Vietnam; 6 General Statistics Office, Hanoi, Vietnam

**Keywords:** Tobacco, Smoking, Hardcore smoker, Socioeconomic inequalities, Vietnam

## Abstract

**Background:**

Despite male smokers being dominant in Vietnam, scarce evidence on trends in socioeconomics inequalities among the hardcore male smokers is available in the country. In this study, we aimed at assessing the trends in socioeconomics inequalities among the hardcore smokers in adult male population in Vietnam over a five-year period from 2010 to 2015.

**Methods:**

We used data from two rounds of the Vietnam Global Adult Tobacco Survey (GATS) conducted in 2010 and 2015. We included only men aged 25 years and above in the analysis. We measured socioeconomic inequalities among hardcore smokers by calculating the concentration index. We conducted multiple logistic regression analysis to identify factors associated with hardcore smoking among men aged 25 years and above.

**Results:**

The results of this study showed that the prevalence of male hardcore smokers aged 25 years and above in Vietnam was 9.5% in 2010 which increased to 13.1% in 2015. The prevalence of male hardcore smokers declined in the richest group from the 2010 level whereas it increased in the middle, poor and poorest groups. All values of weighted concentration indices indicated that the prevalence of male hardcore smokers occurred more among the poor men in Vietnam in both 2010 and 2015. The socioeconomic inequalities in hardcore smokers increased during 2010 and 2015. Residence in urban areas was significantly associated with higher adult male hardcore smoking in our study. Belonging to the age groups between 40 and 59 years, attaining primary and lower education, being self-employed, belonging to the poorest household group, smoking being allowed at home and no rule for smoking at home were associated with higher risk of being hardcore smoker among adult males in Vietnam.

**Conclusions:**

We found increased trends in socioeconomic inequalities in hardcore smoking among the study population. Our study results indicate that existing smoking secession and tobacco control policy and interventions need to be modified or new policies and interventions should be introduced with the perspective of addressing socioeconomic inequalities to have the desired impact. We recommend implementing specific targeted interventions for vulnerable population groups for better results.

## Background

Tobacco use has been a major public health challenge globally as well as in the Southeast Asia region. It is a major risk factor for several non-communicable diseases. It accounts for almost 6 million deaths each year globally [1]. Smoking is a highly prevalent form of tobacco use in Vietnam. As per the Global Adult Tobacco Survey (GATS) 2015 prevalence of current tobacco smoking in adult population in Vietnam is 22.5% with 45.3% in males and 1.1% among females [2].

The data from two rounds of the GATS (2010 and 2015) in Vietnam revealed a slight decrease in prevalence of current smoking from 23.8% in 2010 to 22.5% in 2015 [2, 3]. However, a study based on further in-depth analysis of data showed that this decrease was not statistically significant [4]. Experiences from different countries suggest that light smokers quit smoking relatively faster in comparison with the hardcore smokers who find it difficult to quit smoking. Hardcore smokers are a subgroup of daily smokers with least possibility of quitting and who respond less to tobacco control interventions [5, 6]. Studies in different parts of the world indicate that hardcore smokers are more likely to be males, to be older and to have low levels of education and income [6–9]. A study conducted in Australia showed decline in hardcore smoking in richest two quintiles whereas the hardcore smoking remain stable in poorest two quintiles during 2001 to 2010 [10]. Another study in England showed significant higher trend of higher odds of hardcore smoking with increasing socio-economic deprivation [11]. The difficulties of smoking cessation among hardcore smokers result in low rates of decrease in prevalence of smoking.

The Government of Vietnam has identified tobacco control as one of the priority public health programmes and has taken several steps for controlling the use of tobacco in the country. Some of these steps include signing framework Convention on Tobacco Control (FCTC) in 2003, ratifying it in 2004 as well as introducing National Tobacco Control Policy in government resolution, enacting the first-ever comprehensive Law on Prevention and Control of Tobacco Harms in 2012 starting its effective implementation from 2013 and release of National Strategy on Tobacco Control till 2020 The tobacco control efforts in Vietnam include interventions such as public education, comprehensive ban of tobacco advertising, promotion and sponsorship, restriction on smoking in public places, application of large size graphic health warnings on labels, establishing smoking-free places, increasing tax on tobacco products, raising prices of tobacco products and implementing smoking cessation programmes, ban of tobacco sale for children under 18 and ban of kiddy pack. However, these efforts and interventions seem to produce limited results in reducing the prevalence of current smoking in the country [2, 4]. It calls for further research on various aspects associated with smoking and smokers in the country with special focus on hardcore male smokers as the current smoking prevalence is predominantly high among males in comparison with females.

There is growing evidence in different parts of the world on importance of inequality studies considering the population health [12–14]. Knowledge about the status and trends in socioeconomic inequalities among hardcore smokers is helpful for better planning of future tobacco control interventions [15]. Despite male smokers being dominant in the population of smokers in Vietnam, very little is known about trends in socioeconomics inequalities among the hardcore male smokers in the country. There is a clear need of filling this knowledge and evidence gap in this area. The objective of this study was to assess the trends in socioeconomics inequalities among the hardcore smokers in adult male population in Vietnam over a five-year period from 2010 to 2015.

## Methods

### Data

Data for this study were derived from two rounds of the Vietnam Global Adult Tobacco Survey (GATS) conducted in 2010 and 2015 [3, 4]. The GATS is the global standard that helps to monitor and track tobacco control indicators in countries. Both rounds of the GATS in Vietnam were designed to represent nationally for all non-institutionalized men and women aged 15 years and older. The target population included Vietnamese citizens who mainly resided in Vietnam. The exclusion criteria were the tourists, people living in military-based areas or group quarters, or people residing in hospitals, prisons, nursing homes and other such institutions. The sampling design of the GATS was based on a multi-stage stratified geographically clustered sampling. The total completed interviews were 8996 households in the GATS 2010 (giving the response rate of 95.7%), and 9513 households in the GATS 2015 (giving the response rate of 97.8%). Technical information concerning the study design, sampling and data collection is provided in detail elsewhere [3, 4]. Since the smoking in Vietnam was rare among women (3.6% in 2010, and 1.1% in 2015), we included only men in this analysis. We included only men 25 years old and older because they have reached a stable smoking consumption, so the final sample used in this study consisted of 3554 men in 2010, and 3423 men in 2015.

### Variables

The main outcome variable in this study was the binary variable (yes/no) of hardcore smoker status; specifically, whether or not a man was a hardcore smoker. We defined a hardcore smoker in this study as a current daily smoker, who at the time of survey 1) had been smoking for 5 years or longer, 2) smoked 15 cigarettes per day or more, 3) had made no quit attempt in the past 12 months, and 4) had no intention to stop smoking at all or in the next 12 months. We included age groups, place of residence (urban/rural), education, occupation, ethnicity, marital status, rule regarding smoking at home, and household socioeconomic status as independent variables in the study.

### Measurement of socioeconomic status

A household wealth asset index was used as a proxy for socioeconomic status in this study. The principal components analysis (PCA) was used to construct the household wealth asset index [16]. The durable household assets for this estimation included the binary variable of flush toilet, fixed telephone, cell phone, television, radio, refrigerator, car, motorbike, washing machine, air-conditioner, generator, car/motor boat, computer, and internet connection. The variable with its prevalence smaller than 5% or greater than 95% was excluded from the analysis. We used the threshold of eigenvalues greater than one as criteria for the extraction of PCA, and we used varimax (orthogonal) rotation to improve component interpretation. The household wealth asset scores were divided into five quintiles equivalent to five socioeconomic groups of equal sizes (from the poorest group to the richest group).

### Measurement of socioeconomic inequalities

The socioeconomic inequalities among hardcore smokers were measured by the concentration index. The formula used by us for the calculation of the concentration index was proposed by O’Donnell et al. [17], as follows:

$$ \mathrm{C}=\frac{2}{\upmu}\operatorname{cov}\left(\mathrm{h},\mathrm{r}\right) $$

where μ is the proportion of hardcore smokers in the study population, h is the status of the hardcore smoker of an individual, and r is the fractional rank of individual in the distribution of the socioeconomic status (using the rank of the household asset wealth scores). Thus, the concentration index is the covariance between the hardcore smoker variable (h) and the fraction rank in the socioeconomic status (r). The concentration index varies from – to +1, and at the value of zero, there is no socioeconomic inequality among hardcore smokers in the study population. A negative concentration index indicates that hardcore smokers concentrate more among the poor, while a positive concentration index indicates that hardcore smokers concentrate more among the rich.

### Statistical analysis

All analyses were performed only for men in the general population aged 25 years and above. The prevalence of hardcore smokers with 95% confidence intervals was estimated by the year 2010 and 2015 as well as by age group, place of residence, education, occupation, ethnicity, marital status, rules regarding smoking at home and socioeconomic status. The concentration index was estimated by using the add-in Distributive Analysis Stata Package (DASP) [18]. The concentration index with 95% confidence intervals of each year was estimated, then the change from 2010 to 2015 was compared. We used the probit regression model to standardize the concentration indices and its changes by three methods: 1) Unstandardized, 2) standardized by age, the place of residence, and education, and 3) standardized by all independence variables of the study. We conducted multiple logistic regression analysis to identify factors associated with hardcore smoking among men aged 25 years and above. Sample weights were used in all statistical analyses, and all analyses were performed with STATA 13.1 software. The level of statistical significance was set to 0.05.

## Results

Table 1 shows the weighted number and prevalence of hardcore smokers among men aged 25 years and above in Vietnam in 2010 and 2015. Overall, the prevalence of male hardcore smokers aged 25 years and above in Vietnam was 9.5% (equal to 2,147,516 hardcore smokers in the population) in 2010, and it significantly increased to 13.1% (equal to 3,337,178 hardcore smokers in the population) in 2015. The prevalence of male hardcore smokers in all age groups, except the age group 50–59, increased from 2010 to 2015. The increase in the prevalence of men hardcore smokers in the age groups 25–29 and 40–49 between 2010 and 2015 was more than 5 percentage points. The prevalence of men hardcore smokers residing in rural areas, who had primary or lower education, who were self-employed, who were Kinh ethnicity, who were unmarried and who had smoking allowed at home was significantly higher in 2015 in comparison with that in 2010.

**Table 1:** Weighted number and prevalence of male hardcore smokers aged 25 years and above with 95% CI, Global Adult Tobacco Survey in Vietnam, 2010 and 2015

	Year 2010		Year 2015	
	n	% (95% CI)	n	% (95% CI)
Overall	2,147,516	9.5 (8.3-10.8)	3,337,178	13.1 (11.7-14.5)
Age group (years)				
25-29	116,902	3.8 (2.2-6.2)	303,554	8.9 (5.8-13.4)
30-39	538,977	8.6 (6.7-11.0)	986,740	13.4 (10.9-16.4)
40-49	740,014	11.6 (9.4-14.3)	1,095,262	17.2 (14.4-20.4)
50-59	504,691	14.9 (11.7-18.8)	619,711	14.5 (11.8-17.8)
≥ 60	246,931	7.2 (5.1-10.2)	331,912	8.1 (6.0-10.7)
Residence				
Rural	1,505,777	9.7 (8.2-11.4)	2,300,019	13.7 (11.9-15.7)
Urban	641,739	9.2 (7.7-10.9)	1,037,159	11.9 (10.1-13.9)
Education				
Primary or lower	1,211,413	12 (10.2-14.0)	1,725,433	18.8 (16.4-21.4)
Lower secondary	597,009	8.8 (6.9-11.2)	871,439	12.2 (9.9-14.8)
Upper secondary	256,360	6.8 (4.6-9.8)	467,972	10.6 (7.8-14.1)
College or above	82,734	4.5 (2.9-6.8)	272,335	5.8 (3.9-8.5)
Occupation				
Employed	152,529	4.9 (3.2-7.6)	372,861	6.8 (4.8-9.5)
Self-employed	1,786,126	10.7 (9.2-12.3)	2,721,955	16.5 (14.8-18.4)
No active	208,861	7.7 (5.5-10.7)	242,362	6.8 (4.8-9.7)
Ethnicity				
Minority	337,920	10.6 (7.9-14.0)	535,707	14.0 (10.8-18.1)
Kinh (majority)	1,809,596	9.4 (8.1-10.8)	2,801,471	12.9 (11.5-14.5)
Marital status				
Unmarried	87,622	4.3 (2.3-7.6)	224,774	9.3 (5.9-14.3)
Married	1,989,410	10 (8.7-11.4)	2,988,019	13.5 (12.0-15.1)
Separated/Divorced/Widow	70,483	12 (6.8-20.3)	124,385	13.5 (8.9-20)
Rules regarding smoking at home				
Smoking allowed	420,194	16.2 (12.3-21.1)	871,660	23.0 (19.0-27.6)
No rules	1,511,895	10.3 (8.9-12.0)	1,888,242	14.5 (12.4-16.8)
Not allowed with some exception	142,736	4.2 (2.6-6.8)	382,432	7.7 (5.8-10.3)
Not allowed completely	72,691	3.8 (2.1-6.9)	194,844	5.2 (3.4-7.8)

**Fig. 1:**
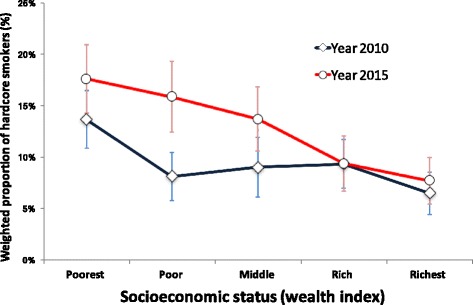
Weighted prevalence of male hardcore smokers aged 25 years and above with 95% CI, Global Adult Tobacco Survey in Vietnam, 2010 and 2015

Figure 1 presents the weighted prevalence of male hardcore smokers and 95% confidence intervals by socioeconomic status in 2010 and 2015. The prevalence of male hardcore smokers in the middle, poorest and poor groups in 2015 was higher than that in 2010. Only the prevalence of male hardcore smokers in the richest group declined in 2015 from 2010.

As shown in Table 2, the weighted concentration indices of male hardcore smokers at age 25 and above were estimated for 2010 and 2015 by three different methods. All values of concentration indices were negative, which indicated that the prevalence of male hardcore smokers occurred more among the poor men in Vietnam in both 2010 and 2015. The changes of the concentration indices from 2010 to 2015 were also negative, indicating that the socioeconomic inequalities increased during 2010 and 2015. The changes of the concentration indices from 2010 to 2015 were statistically significant when using either standardized methods.

**Table 2:** Weighted concentration indices of male hardcore smokers aged 25 years and above with 95% CI, Global Adult Tobacco Survey in Vietnam, 2010 and 2015

Method	Year 2010	Year 2015	Change from 2010 to 2015
	Concentration index (95% CI)		
Unstandardized	-0.124 (-0.190,-0.057)	-0.206 (-0.264,-0.149)	-0.082 (-0.170,0.006)
Standardized by age, residence and education	-0.062 (-0.068,-0.056)	-0.092 (-0.100,-0.084)	-0.030 (-0.041, -0.020)
Standardized by all variables^a^	-0.080 (-0.090,-0.069)	-0.118 (-0.132,-0.103)	-0.038 (-0.056, -0.020)

^a^: age, residence, education, occupation, ethnicity, marital status and rules regarding smoking at home

Table 3 shows the socioeconomic factors associated with hardcore smoking among males aged 25 years and above in Vietnam during 2010 and 2015. The results of multiple logistic regression analysis showed that male hardcore smokers in 2015 had 1.7 times higher odds compared to that in 2010. The significant factors associated to increase the prevalence of men hardcore smokers were in age group 40–49 (odds ratio [OR] = 2.0, 95% confidence interval [CI] =1.3–3.1), age group 50–59 (OR = 2.2, 95% CI =1.4–3.4), living in urban area (OR = 1.4, 95% CI = 1.1–1.7), primary and lower education (OR = 1.9, 95% CI = 1.2–2.9), self-employed (OR = 1.7, 95% CI = 1.2–2.4), belonging to the poorest household group (OR = 1.5, 95% CI = 1.1–2.2). Smoking allowed at home (OR = 4.2, 95% CI = 2.8–6.4) and no rule for smoking at home (OR = 2.7, 95% CI = 1.9–4.0) strongly associated with the increase of the men hardcore smoker’s prevalence.

**Table 3:** Socioeconomic factors associated with male hardcore smokers aged 25 years and above, Global Adult Tobacco Survey in Vietnam, 2010 and 2015: multiple logistic regression

	OR	95% CI
Year		
2010	1	
2015	1.7	1.4-2.0
Age (years)		
25-29	1	
30-39	1.6	1.0-2.5
40-49	2.0	1.3-3.1
50-59	2.2	1.4-3.4
≥ 60	1.1	0.6-1.8
Residence		
Rural	1	
Urban	1.4	1.1-1.7
Education		
Primary or lower	1.9	1.2-2.9
Lower secondary	1.3	0.8-2
Upper secondary	1.2	0.8-2
College or above	1	
Occupation		
Employed	1	
Self-employed	1.7	1.2-2.4
No active	1.1	0.7-1.8
Ethnicity		
Minority	0.8	0.6-1.1
Kinh (majority)	1	
Marital status		
Unmarried	0.8	0.5-1.2
Married	1	
Separated/Divorced/Widow	1.0	0.7-1.5
Rules regarding smoking at home		
Smoking allowed	4.2	2.8-6.4
No rules	2.7	1.9-4.0
Not allowed with some exception	1.4	0.9-2.2
Not allowed completely	1	
Socioeconomic status (Household wealth assets index)		
Poorest	1.5	1.1-2.2
Poor	1.2	0.9-1.8
Middle	1.2	0.8-1.7
Rich	1.1	0.8-1.5
Richest	1	

OR: Odds Ratio

## Discussion

This study examined the trends in socioeconomics inequalities among the hardcore smokers in adult male population in Vietnam over a five-year period from 2010 to 2015. The results of this study show that proportion of male hardcore smokers aged 25 years and above in Vietnam increased to 13.1% in 2015 from 9.5% in 2010. This increased proportion of hardcore male smokers is contrary to the expectations of the impact of tobacco control policies and interventions introduced in Vietnam. Further evidence is required to investigate this substantial growth in hardcore smoking among adult males in Vietnam.

The results of concentration indices showed increasing socioeconomic inequalities in hardcore smoking among the adult male population in Vietnam between 2010 and 2015. This finding indicates towards limited reach and effectiveness of tobacco control and smoking secession interventions in the most disadvantaged populations in the country. These results further reinforce the findings of another study in Vietnam showing a higher prevalence of smoking among disadvantaged groups such as people with lower education and people having less professional occupations [4]. However, contrary to the findings of higher prevalence of smoking among overall population in other studies [2, 4], this study shows higher prevalence of male hardcore smokers among adult male population residing in urban areas. Factors associated with higher prevalence of male hardcore smokers among adults residing in urban areas should be further investigated.

The results of our study showed that the strongest factor associated with increased hardcore smoking was smoking allowed at home which was followed by having no rules regarding smoking at home. The study participants belonging to households where smoking was allowed had 4.2 times higher likelihood of becoming hardcore smoker than the people belonging to households where smoking was not allowed completely. This shows the positive impact of restrictions on smoking at home. Studies in other countries also show that people from households having restriction of smoking are less likely to start or develop smoking habits [19–21]. The household environment is very important source and the most proximal context for people to initiate and continue smoking. Hence, context-specific interventions are required to promote smoking free households.

Other key factors associated with increased hardcore smoking were belonging to the age groups between 40 and 49 and 50–59 years, having primary or low education, being self-employed, belonging to the poorest quintile of the society and residing in urban areas. Age differences in hardcore smoking were found in our study. People in the age group of 50–59 year and in the age group 40–49 year were 2.2 and 2 times more likely of being hardcore smokers compared to people in the age group of 25–29 years. These findings are similar to the findings of other studies in different parts of the world [22–24]. However, the likelihood of being hardcore smoker slightly decreased in the age group of 60 years and above. It may be because of less access to resources and increasing smoking-related health problems among people in this age groups. It could also be due to survival bias as smokers are likely to die earlier.

Our results regarding the higher prevalence of hardcore smoking among adult males with low education levels corroborate the growing evidence on a strong association between education attainment and smoking [4, 23–27]. Our study revealed that people with primary or lower education were 1.9 times more likely to be hardcore smokers than people with college or above education. Our study results also showed consistently decreased chances of being hardcore smoker with increased number of years of education. A significant difference in the odds of being hardcore smokers in the poorest compared to richest quintile was observed in our study indicating poverty as an important gradient for hardcore smoking. This finding is in line with other studies [24, 26, 27].

The results of our study showed that participants belonging to self-employed category had higher odds of hardcore smoking compared to employed and unemployed participants. This finding is in line with other studies [2, 4]. Residence in urban areas was significantly associated with higher adult male hardcore smoking in our study. This finding is contrary to the studies showing the higher prevalence of overall smoking in people residing in rural areas [4, 23, 24]. The reasons behind higher prevalence of hardcore smoking among adult males in Vietnam should be further studied using quantitative and qualitative methods.

This study has some limitations which should be considered while interpreting the results. Both rounds of the GATS survey, the source of data for this study, were based on self-reported information of respondents and no validation of the information provided by them was done from other objective sources. Hence, the self-reported smoking behaviour may not be the true prevalence of hardcore smoking among adult males in Vietnam. Social desirability bias may also be present in our study as the smokers may have exaggerated their intention to quit smoking influenced by the no smoking norm. Survival bias may also be present in our study as it shows no association with age 60 years and above with hardcore smoking. It may be due to survival bias as the smoker are likely to die earlier. Lastly, the GATS surveys used the cross-sectional design, which prevented any interpretation about the causal relationship.

## Conclusion

Our study showed increased prevalence of hardcore smokers among the adult male population in Vietnam over the study period. It also revealed increased trends in socioeconomic inequalities in hardcore smoking among the adult male population. The results of our study have very significant implications for evidence-based programming for smoking cessation and tobacco control in the country. Our study results indicate that smoking secession and tobacco control policy and interventions need to consider socioeconomic inequalities among hardcore male smokers in Vietnam and modify the existing policies and interventions or introduce new policies and interventions with the perspective of addressing socioeconomic inequalities to have the desired impact.

The results of our study also highlight the need of adopting focused approaches for addressing socio-economic inequalities among different population groups of the male hardcore smokers. It is recommended that implementing specific targeted interventions for vulnerable population groups would produce better results. These targeted interventions may include strong communication campaigns on smoke-free homes, stronger enforcement of tobacco control legislation ensuring fines on smoking at the smoke-free places. Since the hardcore smokers have knowledge of smoking hazards but they are unwilling to quit smoking, tobacco consultancy hotline should be expanded with personalized messages indicating the risk to the concerned individuals. Expansion of tobacco cessation services is also recommended along with ensuring community reach to these services.
